# Supplementary material for: Role and mechanism of NCAPD3 in promoting malignant behaviors in gastric cancer
Source: Front Pharmacol. 2024 Apr 22;15:1341039. doi: 10.3389/fphar.2024.1341039 (PMC11070777; doi:10.3389/fphar.2024.1341039)
Supplement: Supplementary file 11 [file DataSheet2.ZIP › GSEA/Oncogenic signature/my_analysis.Gsea.1599461962066/ALK_DN.V1_UP.html]

Details for gene set ALK\_DN.V1\_UP[GSEA]

|  || Dataset | filtered\_dataset.sample\_info.cls#WT\_versus\_NCAPD3\_MUT |
| Phenotype | sample\_info.cls#WT\_versus\_NCAPD3\_MUT |
| Upregulated in class | NCAPD3\_MUT |
| GeneSet | ALK\_DN.V1\_UP |
| Enrichment Score (ES) | -0.60155433 |
| Normalized Enrichment Score (NES) | -2.6697843 |
| Nominal p-value | 0.0 |
| FDR q-value | 0.0 |
| FWER p-Value | 0.0 |
Table: GSEA Results Summary

  

Fig 1: Enrichment plot: ALK\_DN.V1\_UP      
 Profile of the Running ES Score & Positions of GeneSet Members on the Rank Ordered List

  

| SYMBOL | TITLE | RANK IN GENE LIST | RANK METRIC SCORE | RUNNING ES | CORE ENRICHMENT || 1 | 1080 | CFTR | 366 | 0.557 | -0.2199 | No |
| 2 | 6507 | SLC1A3 | 464 | 0.496 | -0.2511 | No |
| 3 | 6237 | RRAS | 869 | -0.280 | -0.5198 | No |
| 4 | 8140 | SLC7A5 | 890 | -0.305 | -0.5104 | No |
| 5 | 10797 | MTHFD2 | 911 | -0.338 | -0.4986 | No |
| 6 | 79094 | CHAC1 | 949 | -0.367 | -0.4966 | No |
| 7 | 5834 | PYGB | 1096 | -0.475 | -0.5646 | Yes |
| 8 | 54541 | DDIT4 | 1133 | -0.502 | -0.5515 | Yes |
| 9 | 639 | PRDM1 | 1159 | -0.523 | -0.5288 | Yes |
| 10 | 3887 | KRT81 | 1168 | -0.536 | -0.4929 | Yes |
| 11 | 23657 | SLC7A11 | 1205 | -0.581 | -0.4736 | Yes |
| 12 | 29968 | PSAT1 | 1215 | -0.589 | -0.4343 | Yes |
| 13 | 440 | ASNS | 1232 | -0.609 | -0.3985 | Yes |
| 14 | 5106 | PCK2 | 1252 | -0.640 | -0.3624 | Yes |
| 15 | 57761 | TRIB3 | 1292 | -0.685 | -0.3372 | Yes |
| 16 | 1116 | CHI3L1 | 1339 | -0.777 | -0.3099 | Yes |
| 17 | 7422 | VEGFA | 1346 | -0.788 | -0.2530 | Yes |
| 18 | 1491 | CTH | 1391 | -1.004 | -0.2066 | Yes |
| 19 | 1475 | CSTA | 1400 | -1.162 | -0.1220 | Yes |
| 20 | 760 | CA2 | 1410 | -1.654 | -0.0000 | Yes |
Table: GSEA details [plain text format]

  

Fig 2: ALK\_DN.V1\_UP      
 Blue-Pink O' Gram in the Space of the Analyzed GeneSet

  

Fig 3: ALK\_DN.V1\_UP: Random ES distribution      
 Gene set null distribution of ES for **ALK\_DN.V1\_UP**
